# Chitin Synthase Genes Are Differentially Required for Growth, Stress Response, and Virulence in *Verticillium dahliae*

**DOI:** 10.3390/jof8070681

**Published:** 2022-06-28

**Authors:** Jun Qin, Peichen Zhao, Ziqin Ye, Lifan Sun, Xiaoping Hu, Jie Zhang

**Affiliations:** 1 State Key Laboratory of Crop Stress Biology for Arid Areas, College of Plant Protection, Northwest A&F University, Yangling 712100, China; qinj@nwafu.edu.cn (J.Q.); xphu@nwsuaf.edu.cn (X.H.); 2 State Key Laboratory of Plant Genomics, Institute of Microbiology, Chinese Academy of Sciences, Beijing 100101, China; gl042563529@126.com (P.Z.); yezq@im.ac.cn (Z.Y.); sunlf@im.ac.cn (L.S.); 3 CAS Center for Excellence in Biotic Interactions, University of Chinese Academy of Sciences, Beijing 100049, China

**Keywords:** *Verticillium*, chitin synthase, virulence, stress

## Abstract

Crop wilt disease caused by *Verticillium dahliae* usually leads to serious yield loss. Chitin, an important component of most fungal cell walls, functions to maintain the rigidity of cell walls and septa. Chitin synthesis mainly relies on the activity of chitin synthase (CHS). Eight *CHS* genes have been predicted in *V. dahliae*. In this study, we characterized the functions of these genes in terms of growth, stress responses, penetration, and virulence. Results showed that *VdCHS5* is important for conidia germination and resistance to hyperosmotic stress. Conidial production is significantly decreased in *Vdchs1*, *Vdchs4*, and *Vdchs8* mutants. *VdCHS1*, *VdCHS2*, *VdCHS4*, *VdCHS6*, *VdCHS7*, and *VdCHS8* genes are important for cell wall integrity, while all mutants are important for cell membrane integrity. All of the *VdCHS* genes, except for *VdCHS3*, are required for the full pathogenicity of *V. dahliae* to *Arabidopsis thaliana* and cotton plants. The in vitro and in vivo penetration of *Vdchs1*, *Vdchs4*, *Vdchs6*, and *Vdchs7* mutants was impaired, while that of the other mutants was normal. Overall, our results indicate that the *VdCHS* genes exert diverse functions to regulate the growth and development, conidial germination, conidial production, stress response, penetration, and virulence in *V. dahliae*.

## 1. Introduction

Chitin is a beta-(1,4) linked polymer of *N*-acetylglucosamine, which is an important structural component of fungal cell walls, but it is absent from plants and vertebrates [1,2,3,4]. Chitin maintains cellular integrity and resistance to environmental stress. The cytoplasmic membrane-bound chitin synthases (CHSs) are the primary enzymes that catalyze chitin chain extension [5]. *CHS* genes from different fungi are divided into three divisions according to their domain architecture [6,7]. Division I consists of classes I, II, and III and division II contains classes IV, V, and VII, whereas division III only consists of class VI.

The *CHS* genes exert different functions in yeast and filamentous fungi. The budding yeast *Saccharomyces cerevisiae* contains three *CHS* genes with diverse functions in cell wall and septum formation, as well as in cell division [8,9,10]. Chs1 acts as a repair enzyme and is responsible for repairing the cell wall of daughter cells after cell division [11]. Chs2 is involved in primary septum formation and cell division [12]. Chs3 is the key enzyme for synthesizing chitin and is responsible for forming the chitin ring at the budding site [13,14]. There are only two chitin synthase genes in fission yeast, *Schizosaccharomyces pombe*, and the *chs1*^+^ gene is involved in ascospore maturation [15]. Another yeast, *Candida albicans*, contains four *CHS* genes (*CaCHSs*). *CaCHS1* is essential for septum formation and lateral cell wall integrity [16]. *CaCHS2* and *CaCHS3* are important for virulence [17,18,19,20] and *CaCHS8* is involved in stress responses [21].

The composition of the *CHS* genes in filamentous fungi is more complex because they usually contain seven to nine *CHS* genes. *Neurospora crassa* contains seven *CHS* genes [22]. *CHS-1* is involved in cell wall biogenesis [23] and *CHS-3* is essential for development [24]. The *CHS-2* RIP (repeat-induced point) mutant *chs-2*^RIP^ does not show defects in germination rate, hyphal elongation, or other morphological phenotypes [25]. While there is no difference in the growth and development between the *chs-4*^RIP^ mutant and the wild type under normal culture conditions, *CHS4* is involved in supplementing chitin syntheses under various growth conditions [26]. Six *CHS* genes have been isolated from *Aspergillus nidulans*, two of which (*CsmA* and *CsmB*) contain an N-terminal myosin motor-like domain and are essential for hyphal tip growth [27,28]. Both *CsmA* and *CsmB* deletion mutants are defective in balloon and intrahyphal hyphae formation and hyphal lysis under low osmotic conditions [27,29]. All of the *ChsA*, *ChsB*, and *ChsC* genes play roles in septa synthesis and conidial development [30,31]. The expression of *ChsE* is significantly upregulated according to osmotic stress.

*Verticillium dahliae* is a soil-borne fungal pathogen that infects a broad range of crops, causing wilt and, thus, yield reduction and billions of dollars’ worth of economic loss [32]. Its hosts range from cotton, potato, eggplant, sunflower, strawberry, redbud, smoke tree, and so on [32,33,34,35]. *V. dahliae* may survive in the soil for more than 14 years in the form of microsclerotia [36]. Under favorable soil moisture conditions, the microsclerotia germinate to hyphae and directly penetrate the host roots and colonize the xylem vessels [37,38], causing symptoms such as foliar wilting, chlorosis, plant stunting, vascular browning, and even death [39,40]. Due to the long-lived microsclerotia and residing vascular bundle of the pathogen, *Verticillium* wilt is difficult to control. Thus, characterizing the virulent genes in *V. dahliae* could provide fungal-specific targets for applicability in engineering crop resistance.

The functions of the *CHS* genes regarding the regulation of the development and pathogenicity of *V*. *dahliae* remain unclear. Here, we systematically characterized the functions of the *CHS* genes in the *V*. *dahliae* strain V592. The genome of V592 encodes eight putative *CHS* genes. We generated gene deletion mutants for each of the predicted *CHS* genes and examined their development and pathogenicity. Our findings revealed that the *VdCHS* genes function differently in regulating the growth, development, penetration, and pathogenicity of *V. dahliae*.

## 2. Materials and Methods

### 2.1. Fungal Strains, Plant Materials, and Growth Conditions

The *V*. *dahliae* strain V592 [41] was used as the wild-type strain in this study. Typically, the strains of V592 and all of the *CHS* gene deletion mutants were grown on a potato dextrose agar (PDA) medium at 25 °C in the dark. A PDA medium with additions of 0.2 mg/mL Congo red, 0.7 M NaCl, 50 μg/mL calcofluor white, 1.2 M sorbitol, or 0.01% SDS as indicated was used for stress response assays. Eighteen-day-old PDA plates were used for the measurement of conidial production. Conidia harvested from a 5-day-old potato dextrose broth (PDB) medium were resuspended to 10^5^/mL and spread on a PDA medium for 2 days to detect the germination rate. The conidia of 10^5^ were used for penetration assays. Typically, the conidia were harvested from a 5-day PDB liquid culture and adjusted to 10^7^/mL in sterile ultra-pure water, then a 10 μL conidia suspension was inoculated on minimal medium covered with a layer of cellophane membrane. After growing the culture for 3 days at 25 °C in the dark, the cellophane membrane was removed and the plates were left to grow for another 3 days before photographs were taken [42]. Conidia of 10^7^/mL and 10^6^/mL were used to infect *Arabidopsis thaliana* and cotton plants, respectively.

A two-week-old *Arabidopsis* col-0 and three-week-old cotton plants “Xinluzao No. 16” [43] were used for fungal infection assays. *A. thaliana* was grown in a greenhouse under a 16 h light/8 h dark cycle at 22 ± 1 °C. Cotton plants were grown in a greenhouse under a 16 h light/8 h dark cycle at 25 ± 1 °C.

### 2.2. Generation of Eight CHS Deletion Mutants and Their Complementation Strains

The upstream and downstream flanking sequences of the target *CHS* genes were PCR amplified from the genomic DNA of V592 and inserted into the pGKO-HPT binary vector [44]. The resulting vectors were then transformed into the *Agrobacterium tumefaciens* strain EHA105. The conidia of V592 were co-cultured with the corresponding *A. tumefaciens* strains to generate the *CHS* deletion mutants via *A. tumefaciens*-mediated transformation (ATMT). The *CHS* deletion mutants were cultured on PDA plates containing 50 mg/mL hygromycin (Roche, Basel, Switzerland) and 50 mg/mL 5-fluoro-2′-deoxyuridine (Sigma, St. Louis, MO, USA) and identified by PCR and Southern blot hybridizations. For generating complementation strains, the open reading frame of each *CHS* gene was PCR amplified from the genomic DNA of V592 and inserted into the pNat-Tef-GFP vector [43]. The resulting vectors were then transformed into the *A*. *tumefaciens* strain EHA105. The conidia of each *CHS* deletion mutant were co-cultured with the corresponding *A. tumefaciens* strains to generate the complementation strains for the *CHS* deletion mutants via ATMT. The complementation strains were cultured on PDA plates containing 50 mg/mL hygromycin and 50 mg/mL geneticin and identified by PCR. All primers used are listed in Table S1.

### 2.3. Southern Blot Hybridizations

The genomic DNA of V592 and all the *CHS* deletion mutants was isolated and subjected to Southern blot analyses using the DIG High Prime DNA Labelling and Detection Starter Kit I (Roche), following the instructions provided by the manufacturer. Briefly, 20 μg of genomic DNA from V592 and all of the *CHS* mutants was isolated and digested with the indicated restriction enzymes (HindIII and NcoI for *Vdchs1*, HindIII and BamHI for *Vdchs2*, BamHI for *Vdchs3*, KpnI and PstI for *Vdchs4*, ApaI for *Vdchs5*, KpnI and PacI for *Vdchs6*, BsaI for *Vdchs7*, EcoRI and XhoI for *Vdchs8*). The digested DNA was separated by electrophoresis in an agarose gel and transferred onto a nylon membrane. The nylon membrane was used for hybridization and developing.

### 2.4. Plant Infection Assay

Two-week-old col-0 seedlings were used to assess the pathogenicity of each *CHS* mutant. Conidia were harvest from a PDB medium and adjusted to 10^7^/mL in sterile water. The roots of the two-week-old *Arabidopsis* seedlings were soaked into the conidia suspension and stayed there for 30 min before being replanted into the soil. For the cotton infection assays, conidia of 10^6^/mL were used to inoculate three-week-old cotton seedlings, as described [41]. The disease grade was classified into four grades as follows: Grade 0 (no symptoms), Grade 1 (0–25% wilted leaves), Grade 2 (25–50% wilted leaves), Grade 3 (50–75% wilted leaves) and Grade 4 (75–100% wilted leaves). The symptoms of each strain were investigated at 21 dpi and indicated by the disease index [45].

### 2.5. Fluorescence Microscopy

Three-week-old cotton plants were inoculated with 10^6^/mL of conidia from GFP-labeled *CHS* deletion strains. The lateral roots of the infected cotton were isolated and sliced to be visualized by a SP8 laser confocal scanning microscopy system three days post-inoculation. FM4-64 was used to stain the plasma membrane of the cotton cells.

## 3. Results

### 3.1. Identification of Eight Putative Chitin Synthase Genes in Verticillium Dahliae

**Figure 1:**
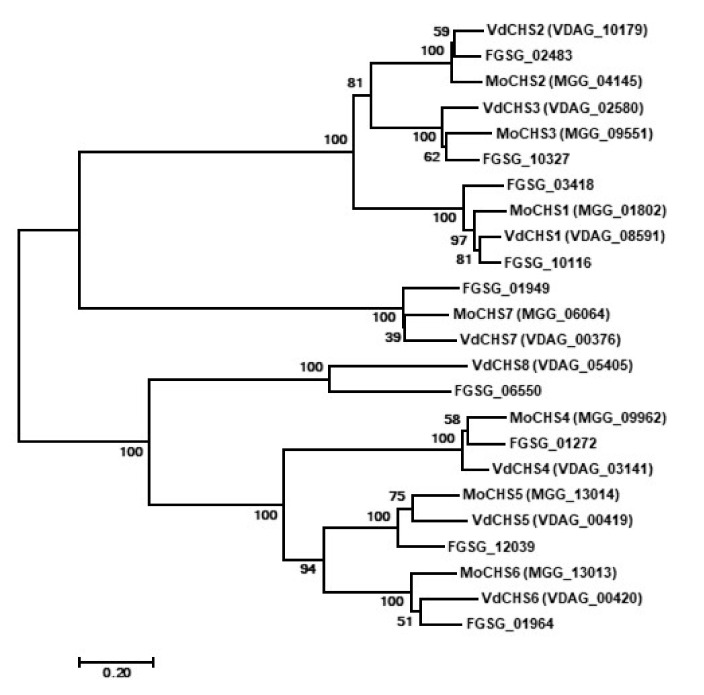
Phylogenetic analysis of *CHS* genes in *Verticillium dahliae*, *Magnaporthe oryzae*, and *Fusarium graminearum*. The full protein sequences of the *CHS* genes in *V*. *dahliae*, *M*. *oryzae*, and *F*. *graminearum* were downloaded from NCBI (https://www.ncbi.nlm.nih.gov/, accessed on 25 March 2019) and aligned using the MAGA 9.0 software. An unrooted phylogenetic tree was constructed by the Neighbor-Joining method based on the alignment of the full protein sequences of the *CHS* genes in *V*. *dahliae*, *M*. *oryzae*, and *F*. *graminearum*. The number of bootstrap replications was set to 1000. The numbers on the branches indicate the bootstrap value for each branch and the bar indicates 0.2 distance units.

There are eight predicted *CHS* genes in *V. dahliae*. Based on phylogenetic analysis with *M. oryzae* and *F*. *graminearum* *CHS* genes, the putative *V. dahliae CHS* genes were designated as *VdCHS1* (*VDAG_08591*), *VdCHS2* (*VDAG_10179*), *VdCHS3* (*VDAG_02580*), *VdCHS4* (*VDAG_03141*), *VdCHS5* (*VDAG_00419*), *VdCHS6* (*VDAG_00420*), *VdCHS7* (*VDAG_00376*), and *VdCHS8* (*VDAG_05405*) [46], according to their identity at the amino acid level (Figure 1). The *V. dahliae* and *F*. *graminearum* genomes encode a new class of *CHS* gene named *CHS8* [47], which is absent from the *M. oryzae* genome [48].

### 3.2. Generation of CHS Gene Deletion Mutants

**Figure 2:**
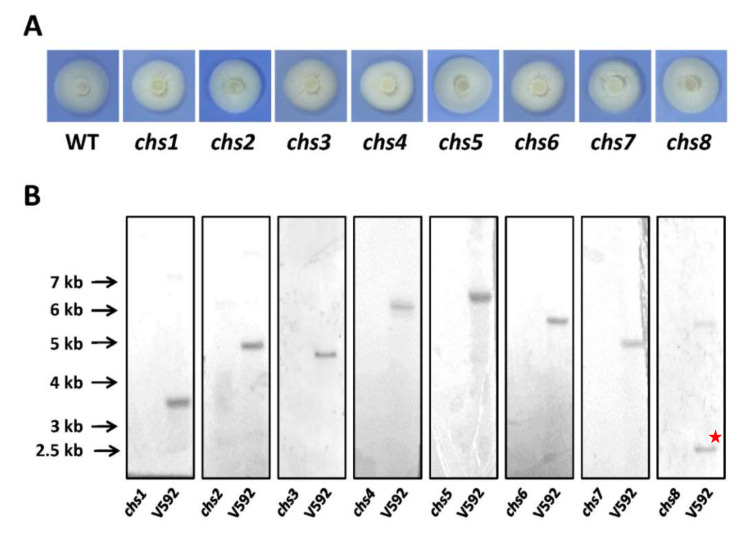
Colony morphology and Southern blot analyses of the eight *CHS* deletion mutants in *V. dahliae*. (**A**) The eight *CHS* deletion mutants were generated and cultured on PDA plates at 25 °C in dark for 7 days to examine the colony morphology. The experiments were repeated five times with similar results. (**B**) Southern blot analyses of the eight *CHS* gene deletion mutants. The genomic DNA of V592 and *CHS* deletion mutants was isolated and digested by the corresponding restriction enzymes. The full-length genomic DNA of each gene was used as a probe to be labeled by an isotope.

To systematically characterize the functions of these *CHS* genes in *V. dahliae*, each *VdCHS* gene was individually knocked-out in the wild type (WT) V592 (Figure 2A). All gene replacement constructs were generated using the pGKO-HPT binary vector (Wang et al., 2016) and transformed into V592. The resulting mutant strains were identified by PCR and Southern blot hybridizations (Figure 2B). When cultured on a PDA plate, all *VdCHS* deletion mutants exhibited similar growth rates to that of V592 (Figure 2A and Table 1). The *Vdchs1*, *Vdchs3*, *Vdchs6*, and *Vdchs7* mutants presented a winkled surface morphology (Figure 2A).

We next examined conidiations in the *VdCHS* deletion mutants. Although a few conidia were produced, the conidiation in the *Vdchs1*, *Vdchs4*, and *Vdchs8* mutants was significantly impaired when they were cultured on PDA plates (Table 1). The conidia produced by V592 are ~3000 times higher than those of *Vdchs1* and *Vdchs8* mutants and ~300 times higher than those of the *Vdchs4* mutant. However, the difference in conidiation between V592 and the other mutants was not beyond an order of magnitude (Table 1).

### 3.3. VdCHS5 Regulates Germination and Tolerance to Hyperosmotic Stress

Conidia germination is a crucial step for establishing successful infections in host plants. Thus, we also examined the conidial germination ability of the *VdCHS* deletion mutants. The conidia of most *VdCHS* deletion mutants retained a normal germination ability compared with the WT. However, the deletion of the *VdCHS5* gene reduced the germination rate to about half of that in the WT (Table 1), suggesting a possible reduced pathogenicity of the *Vdchs5* mutant in host plants.

**Figure 3:**
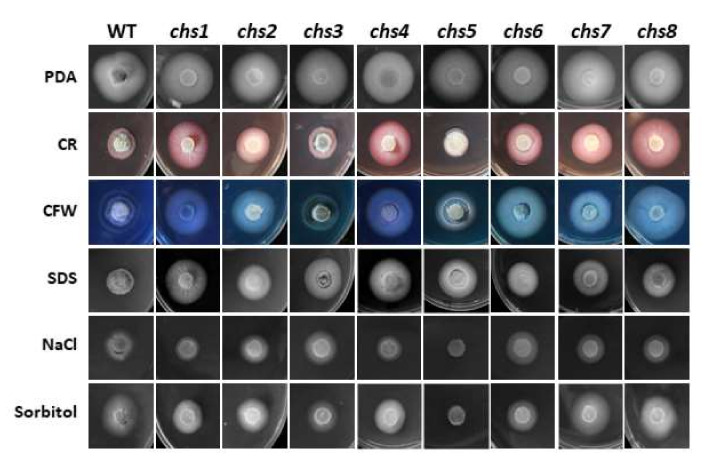
The stress responses of the eight *CHS* gene deletion mutants in *V. dahliae*. The V592 and the eight *CHS* gene deletion mutants were cultured on a PDA agar medium with an addition of 0.2 mg/mL Congo red (CR), 50 μg/mL calcofluor white (CFW), 0.01% SDS, 0.7 M NaCl, or 1.2 M Sorbitol, respectively. After a 7-day growth period at 25 °C in the dark, growth diameters were measured. The experiments were repeated three times with three plates each time and similar results were obtained.

To test whether the *VdCHS* genes are involved in regulating responses to hyperosmotic stress, each *VdCHS* gene deletion mutant was cultured on PDA plates with an addition of 0.7 M NaCl or 1.2 M Sorbitol. The results showed that the *Vdchs5* mutant was hypersensitive to hyperosmotic stress and the growth rate was considerably reduced on both the 0.7 M NaCl and 1.2 M Sorbitol culture media (Figure 3 and Table 2). The *Vdchs1* and *Vdchs4* mutants were more sensitive to 0.7 M NaCl than the WT V592, while *Vdchs3* and *Vdchs6* mutants were more sensitive to 1.2 M Sorbitol than the WT V592 (Figure 3 and Table 2). Thus, the *VdCHS5* gene is essential for a response to hyperosmotic conditions. The *VdCHS1*, *VdCHS3*, *VdCHS4*, and *VdCH6* genes are involved in tolerance to hyperosmotic conditions.

### 3.4. Most VdCHS Genes Function in Cell Wall Maintenance or Cell Membrane Integrity

The ability to resist environmental stress and maintain cell wall and cell membrane integrity is important for the pathogenesis of plant pathogenic fungi. To investigate the regulatory functions of the *VdCHS* genes in cell wall and cell membrane integrity, these mutants together with the WT V592 were cultured on PDA plates with an addition of Congo red, calcofluor white, or Sodium dodecyl sulfate (SDS), respectively. Most *VdCHS* deletion mutants were less sensitive to the 0.2 mg/mL Congo red and 50 μg/mL calcofluor white than the WT, except for the *Vdchs3* and *Vdchs5* mutants (Figure 3 and Table 2). These results indicate that *VdCHS1*, *VdCHS2*, *VdCHS4*, *VdCHS6*, *VdCHS7*, and *VdCHS8* genes function in cell wall integrity. All *CHS* deletion mutants were less sensitive to the 0.01% SDS than the WT, indicating that all *VdCHS* genes are required for maintaining a cell membrane integrity.

### 3.5. All VdCHS Gene Deletion Mutants, except for Vdchs3, Are Compromised during Plant Infection

To examine whether the *VdCHS* genes are required for pathogenicity, each *VdCHS* gene deletion mutants was root-inoculated into *Arabidopsis thaliana* and cotton (*Gossypium hirsutum*) plants and assayed for disease indices. Except for the *Vdchs3* mutant, all mutants exhibited a significant reduced pathogenicity in *Arabidopsis* and cotton plants compared with that of the WT (Table 1). The cotton plants inoculated with the wild type or the *Vdchs3* mutant developed chlorosis and defoliation at 21 days post-inoculation, while the other mutants only exhibited slight chlorosis. The *Arabidopsis* also showed milder symptoms when inoculated with other mutants, except for *Vdchs3*, compared with the wild type. These results indicate that the *VdCHS* genes are vital for the pathogenicity of *V. dahliae*.

### 3.6. Four VdCHS Gene Deletion Mutants Have Impaired Penetration

**Figure 4:**
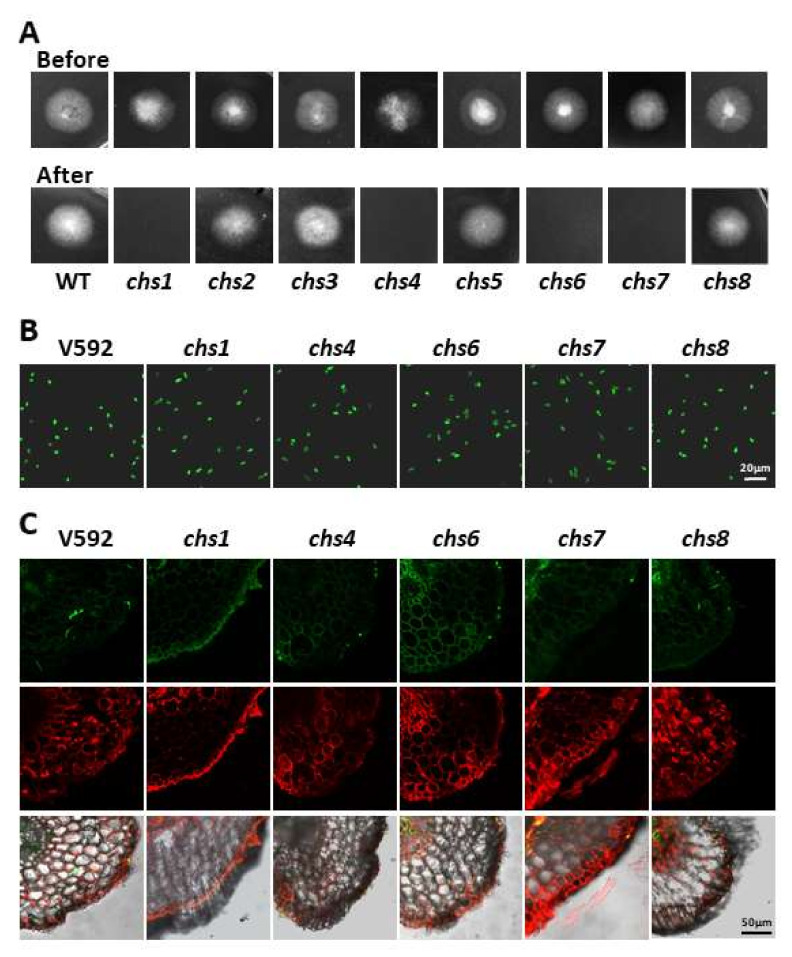
Penetration of the *CHS* gene deletion mutants in vitro and in vivo. (**A**) Growth of the *CHS* gene deletion mutants before and after the removal of the cellophane membrane. The V592 and the *CHS* gene deletion mutants were cultured on minimal medium overlaid with a cellophane layer for 3 days. The cellophane membranes were removed and the plates were cultured for another 3 days. Photographs were taken just before and 3 days after the membranes’ removal. (**B**) GFP-labelled conidia of the V592 and the *CHS* gene deletion mutants. (**C**) Colonization of the *CHS* gene deletion mutants in the root vascular bundles of the cotton. The related *CHS* gene deletion mutants were GFP-labeled and inoculated the cotton roots. Cross sections of the infected cotton roots were visualized by laser confocal scanning microscopy 5 days post-inoculation. The experiments were repeated three times with at least twenty slices of the roots each time; similar results were obtained. The plasma membrane of the cotton roots was stained red with FM4-64.

To examine whether the reduced pathogenicity is attributed to a deficiency in the penetration, all of the *VdCHS* gene deletion mutants were subjected to in vitro penetration assays. The WT and mutant strains were cultured on an MM agar medium covered with a cellophane membrane for 3 days. After the cellophane membrane was removed, the medium was cultured for another 4 days. Fungal hyphae were observed to have penetrated the cellophane membrane for the WT V592, *Vdchs2*, *Vdchs3*, *Vdchs5*, and *Vdchs8* mutants but not for the *Vdchs1*, *Vdchs4*, *Vdchs6*, or *Vdchs7* mutants (Figure 4A). These results suggested that the *VdCHS1*, *VdCHS4*, *VdCHS6*, and *VdCH7* genes are required for penetration.

The *Vdchs1*, *Vdchs4*, *Vdchs6*, and *Vdchs7* mutant strains were further assessed for their ability to penetrate cotton plants. These mutant strains and the WT V592 were labeled with a green fluorescent protein (GFP) (Figure 4B) and incubated with cotton roots for 3 days. Cross sections of the incubated cotton roots were examined for the presence of GFP-labeled *V. dahliae* hyphae. Consistently with the results of the in vitro penetration assays, the fluorescence imaging assays indicated the presence of GFP-labeled hyphae for V592, but not for the mutant strains, in the vascular bundle of the cotton roots (Figure 4C). These results indicate that the *VdCHS1*, *VdCHS4*, *VdCHS6*, and *VdCH7* genes are required to successfully penetrate the host plants. Thus, the reduced pathogenicity of the *Vdchs1*, *Vdchs4*, *Vdchs6*, and *Vdchs7* mutant strains is likely to be attributed to a defective penetration.

### 3.7. The VdCHS2, VdCHS5, and VdCHS8 Genes Likely Contribute to Post-Penetration Virulence

**Figure 5:**
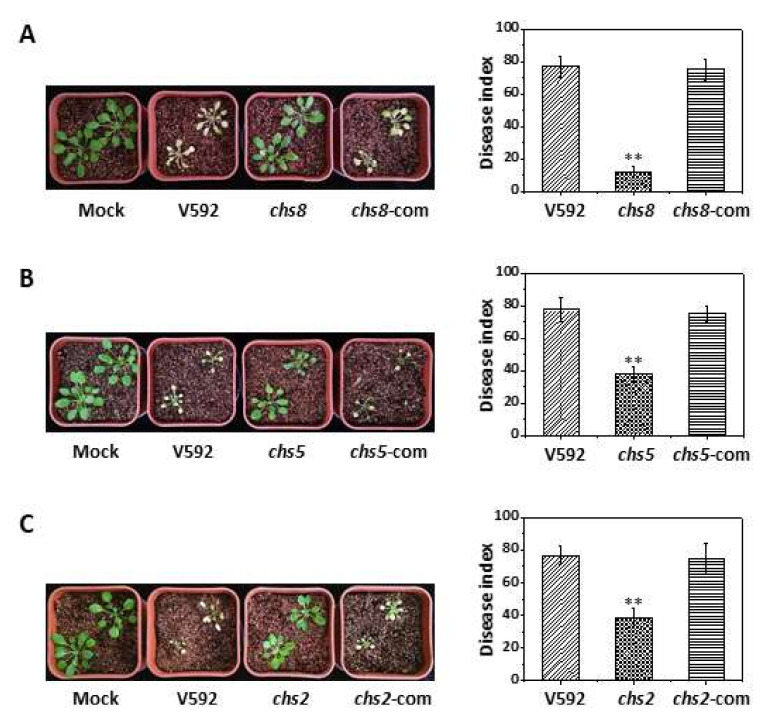
Pathogenicity of the *Vdchs2*, *Vdchs5*, and *Vdchs8* mutants. Two-week-old *Arabidopsis thaliana* seedlings were infected by the three penetration-unaffected mutants *chs2* (**A**), *Vdchs5* (**B**), and *Vdchs8* (**C**), as well as their complementation strains. All of the three complementation strains recovered the defect in the pathogenicity of the corresponding mutants. ** indicates a significant difference at a *p*-value of <0.01.

To further confirm that *VdCHS2*, *VdCHS5*, and *VdCHS8* are required for the *V. dahliae* pathogenicity, complemented strains were generated for the *Vdchs2*, *Vdchs5*, and *Vdchs8* mutants and examined for virulence in *Arabidopsis* plants. As shown in Figure 5, the virulence of the *Vdchs2*, *Vdchs5*, and *Vdchs8* mutants was restored upon the complementation of the corresponding *VdCHS* genes (Figure 5), indicating that *VdCHS2*, *VdCHS5*, and *VdCHS8* are required for the full pathogenicity of *V. dahliae*. As the *Vdchs2*, *Vdchs5*, and *Vdchs8* mutants exhibited an intact penetration ability, the reduced pathogenicity of these mutant strains could be attributed to a post-penetration virulence.

## 4. Discussion

There are seven classes of *CHS* genes belonging to three divisions [7]. Many *CHS* genes are involved in regulating virulence in plant pathogenic filamentous fungi. In this study, we characterized the functions of eight predicted *VdCHS* genes in the *V. dahliae* development and pathogenicity. All of the *VdCHS* deletion mutants, except for the *Vdchs3* mutant, exhibited a defective pathogenicity in plants. The *Vdchs5* mutant was defective in conidia germination and resistance to hyperosmotic stress. A deletion of the *VdCHS1* or *VdCHS8* genes significantly impaired the conidia production, while deletion of the *VdCHS1*, *VdCHS4*, *VdCHS6*, or *VdCHS7* impaired the ability of *V*. *dahliae* to penetrate a cellophane membrane and cotton roots. *VdCHS2*, *VdCHS5*, and *VdCHS8* are required for the full pathogenicity of *V*. *dahliae*, but not for penetration.

The *F. graminearum* genome encodes nine *CHS* genes, which regulate mycelial growth, virulence, or stress response [3,47,49,50]. Deletion of the *FgCHS2* gene causes defects in the growth and virulence, while deletion of the *FgCHS3b* gene is lethal [49]. The *FgCHS8* gene is involved in stress responses, deoxynivalenol production, and virulence [47]. Deletion of the *FgCHS2*, *FgCHS5*, or *FgCHS7* gene greatly compromises the *F. graminearum* pathogenicity in barley heads [49,50]. The genome of *M. oryzae* encodes seven *CHS* genes (*MoCHS1* to *MoCHS7*). *MoCHS1*, *MoCHS6*, and *MoCHS7* are required for the full pathogenicity in barley and rice seedlings [48,51]. Moreover, *MoCHS6* and *MoCHS7* are essential for appressorium penetration and invasive growth on rice leaf sheaths and barley leaves [48]. The pathogen of the grey mold disease *Botrytis cinerea* is a filamentous ascomycete fungus that contains eight *CHS* genes (*Bcchs*) [6]. Among them, *Bcchs1*, *Bcchs3a*, *Bcchs6*, and *Bcchs7* are required for the full pathogenicity of *B. cinerea* [52,53,54]. The *Bcchs5* gene regulates the stress response to cell wall damage [55]. These results suggest differential functions of the *CHS* genes in pathogenicity among different fungi.

The *Vdchs2*, *Vdchs5*, and *Vdchs8* mutants exhibited an intact penetration ability but a reduced pathogenicity, raising the possibility of post-penetration virulence functions of *VdCHS2*, *VdCHS5*, and *VdCHS8*. Interestingly, the deletion of *Bcchs7* resulted in a reduced virulence in *Phaseolus vulgaris* and *Arabidopsis thaliana*, but a normal virulence in *Vitis vinifera* [56], suggesting host-dependent functions or post-penetration functions of the *CHS* genes in virulence.

The *M. oryzae Mochs1* and *Fusarium asiaticum Fachs1* mutants decreased conidiation by 81.3% and 22%, respectively, compared with that of the WT [48,57]. Our results showed that the conidiation had reduced by 99.9% in the *Vdchs1* mutant, implying a similar function of *CHS1* for regulating conidiation in these strains. The *Mochs1* and *Fachs1* mutants exhibit a defect in conidial germination [48,57]. However, conidial germination in the *Vdchs1* mutant was comparable to that of the WT. Instead, the *Vdchs5* mutant was defective in conidial germination (Table 1). Some *CHS* genes are required for the growth rate in *M. oryzae* and other fungi [48]. Mutations of any *VdCHS* genes did not affect the hyphal growth rate of *V. dahliae*.

Disruption of the *CHS* genes results in a significantly decreased pathogenicity in *M. oryzae*, *F. graminearum*, *B*. *cinerea*, and *V. dahliae* [48,50,53], indicating that the *CHS* genes may have the potential to be conserved inhibitory targets for engineering plant resistance [58].

## Figures and Tables

**Table 1:** A phenotype characterization of the *CHS* deletion mutants in *V. dahliae.*

	Growth Rate (mm/Day) ^1^	Germination (%) ^2^	Conidiation (10^6^ Spores/ Plate) ^3^	Disease Index on *Arabidopsis* ^4^	Disease Index on Cotton ^5^
V592 (WT)	1.9 ± 0.1 ^a,^*	97.0 ± 4.5 ^a^	1117.5 ± 502.6 ^b^	67.7 ± 5.5 ^a^	60.5 ± 3.3 ^b^
*chs1*	1.8 ± 0.1 ^a^	93.0 ± 6.1 ^a^	0.4 ± 0.4 ^e^	26.0 ± 2.8 d^e^	17.1 ± 2.0 ^f^
*chs2*	1.8 ± 0.1 ^a^	98.0 ± 2.6 ^a^	535 ± 487.7 ^c^	16.7 ± 3.8 ^f^	3.9 ± 1.9 ^h^
*chs3*	1.8 ± 0.1 ^a^	95.5 ± 5.2 ^a^	476.7 ± 77.7 ^cd^	59.4 ± 5.5 ^b^	66.1 ± 5.0 ^a^
*chs4*	1.8 ± 0.1 ^a^	90.5 ± 7.4 ^a^	38.2 ± 32.2 ^de^	16.3 ± 3.2 ^f^	22.7 ± 3.5 ^e^
*chs5*	1.8 ± 0.1 ^a^	44.0 ± 6.1 ^b^	1823.3 ± 402.5 ^a^	42.7 ± 5.8 ^c^	31.2 ± 2.8 d
*chs6*	1.9 ± 0.1 ^a^	94.5 ± 5.2 ^a^	210 ± 166.4 ^cde^	13.9 ± 3.3 ^f^	11 ± 3.4 ^g^
*chs7*	1.9 ± 0.1 ^a^	96.5 ± 3.8 ^a^	137 ± 40.8 ^cde^	32.6 ± 6.1 d	36.3 ± 2.6 ^cd^
*chs8*	1.9 ± 0.1 ^a^	99.0 ± 2.0 ^a^	0.3 ± 0.2 ^e^	18.4 ± 5.0 ^ef^	37.9 ± 3.5 ^c^

^1^ Average daily extension length of the fungal colony on PDA plates. ^2^ Percentage of conidia geminated after a 12 h culture on PDA plates covered with a layer of cellophane membrane. Ten visual fields were analyzed for each strain. ^3^ Eighteen-day-old PDA plates were used to count the number of conidia produced. ^4^ Disease indices on *Arabidopsis* of *CHS* deletion mutants were measured with 24 *Arabidopsis* seedlings 21 days post-inoculation. ^5^ Disease indices on cotton plants of *CHS* deletion mutants were measured with 24 cotton seedlings 21 days post-inoculation. * Means and standard deviations were calculated using results from three independent experiments. Data were analyzed with the SPSS one-way ANOVA method. Different Greek letters denote statistically significant differences. (*p* = 0.05).

**Table 2:** Stress responses on the vegetative growth of the *CHS* deletion mutants.

	0.7 M NaCl	1.2 M Sorbitol	0.01% SDS	50 μg/mL CFW	0.2 mg/mL Congo Red
V592 (WT)	0.39 ± 0.04 ^bc,^*	0.69 ± 0.06 ^ab^	0.55 ± 0.02 ^c^	0.71 ± 0.05 ^e^	0.40 ± 0.03 ^f^
*chs1*	0.25 ± 0.02 ^d^	0.48 ± 0.01 ^de^	0.83 ± 0.04 ^a^	0.77 ± 0.07 ^cde^	0.87 ± 0.04 ^b^
*chs2*	0.36 ± 0.02 ^c^	0.52 ± 0.07 ^cde^	0.77 ± 0.06 ^ab^	0.85 ± 0.02 ^c^	0.69 ± 0.01 ^cd^
*chs3*	0.45 ± 0.03 ^a^	0.42 ± 0.04 ^ef^	0.73 ± 0.13 ^ab^	0.68 ± 0.10 ^e^	0.49 ± 0.07 ^e^
*chs4*	0.27 ± 0.06 ^d^	0.54 ± 0.02 ^cd^	0.78 ± 0.03 ^ab^	0.82 ± 0.02 ^cd^	0.83 ± 0.03 ^b^
*chs5*	0.00 ± 0.00 ^e^	0.11 ± 0.02 ^g^	0.86 ± 0.03 ^a^	0.74 ± 0.02 ^de^	0.35 ± 0.02 ^g^
*chs6*	0.42 ± 0.03 ^ab^	0.37 ± 0.03 ^f^	0.68 ± 0.02 ^b^	0.98 ± 0.07 ^b^	0.71 ± 0.02 ^c^
*chs7*	0.35 ± 0.01 ^c^	0.73 ± 0.05 ^a^	0.75 ± 0.05 ^ab^	0.75 ± 0.08 ^de^	0.63 ± 0.05 ^d^
*chs8*	0.34 ± 0.03 ^c^	0.60 ± 0.12 ^bc^	0.66 ± 0.01 ^b^	1.10 ± 0.03 ^a^	0.98 ± 0.02 ^a^

The V592 and the eight *CHS* deletion mutants were cultured on a PDA agar medium with an addition of 0.7 M NaCl, 1.2 M sorbitol, 0.01% SDS, 50 μg/mL calcofluor white (CFW), or 0.2 mg/mL Congo red, respectively. The diameter of the colonies was measured 5 days after inoculation. The colony extending percentage (%) = (the diameter of the indicated stress cultures/the diameter of the regular PDA cultures) × 100%. * Means and standard deviations were calculated using the results from three independent experiments. Data were analyzed with the SPSS one-way ANOVA method. Different Greek letters denote statistically significant differences (*p* = 0.05).

## Data Availability

The data presented in this study are available within the article and the supplementary materials.

## Supplementary Materials

The following supporting information can be downloaded at: https://www.mdpi.com/article/10.3390/jof8070681/s1, Figure S1: The amino-acid alignment of eight chitin synthases in *Verticillium dahliae*; Figure: S2: PCR confirmation of eight *VdCHS* deletion mutants; Table S1: Primers used in this study.
