# Supplementary material for: Chitin Synthase Genes Are Differentially Required for Growth, Stress Response, and Virulence in Verticillium dahliae
Source: J Fungi (Basel). 2022 Jun 28;8(7):681. doi: 10.3390/jof8070681 (PMC9320267; doi:10.3390/jof8070681)
Supplement: Supplementary file 1 [file jof-08-00681-s001.zip › Supplementary Figures andTable/Fig S2 final.pdf]

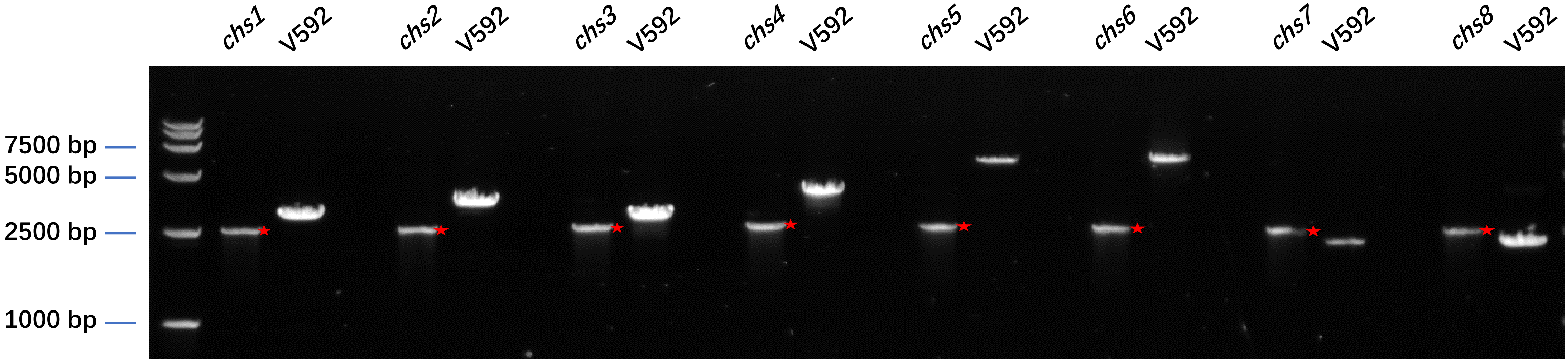

Figure S2. PCR confirmation of eight *VdCHS* deletion mutants. Primers in 5′ and 3′ Flanking sequences of each gene (Table S1) were used to PCR amplified either *VdCHS* gene in the WT or the hygromycin resistance gene (★) in the mutants.
