# Supplementary material for: Chitin Synthase Genes Are Differentially Required for Growth, Stress Response, and Virulence in Verticillium dahliae
Source: J Fungi (Basel). 2022 Jun 28;8(7):681. doi: 10.3390/jof8070681 (PMC9320267; doi:10.3390/jof8070681)
Supplement: Supplementary file 1 [file jof-08-00681-s001.zip › Supplementary Figures andTable/Table S1 revised.pdf]

Table S1. PCR primers used in this study.

**Primer**	**sequence (5'-3')**
VDAG_00419-up-F	GGGTTTAAUCAAGCGAAGACGATAGGG
VDAG_00419-up-R	GGACTTAAUGATGGCGGATAAGATCAGGT
VDAG_00419-down-F	GGCATTAAUCGTAACCTCTGCACTCCA
VDAG_00419-down-R	GGTCTTAAUGGAAATGGCGAGCAGAAC
VDAG_00420-up-F	GGGTTTAAUGCAGGTCTTGCCCTTTAT
VDAG_00420-up-R	GGACTTAAUGGGACTTGAAGCAGACGA
VDAG_00420-down-F	GGCATTAAUTTTGAAACCCGACATTTACG
VDAG_00420-down-R	GGTCTTAAUCCCTGTTCGCTTCCCACT
VDAG_02580-up-F	GGGTTTAAUAGAGGGAACTCGTGTGTTTC
VDAG_02580-up-R	GGACTTAAUCGTACGCGGTCGTTGTAG
VDAG_02580-down-F	GGCATTAAUAGAGACACAATCGCAAGGG
VDAG_02580-down-R	GGTCTTAAUTCGCCAAAACGGAAAACT
VDAG_03141-up-F	GGGTTTAAUCCATCCACCCATCCATCT
VDAG_03141-up-R	GGACTTAAUCGGTCCTGCGGAAGTAAC
VDAG_03141-down-F	GGCATTAAUGTGTTTTTATGACGGGTGGT
VDAG_03141-down-R	GGTCTTAAUTTGCGAGGGTGAAGACTGT
VDAG_08591-up-F	GGGTTTAAUCAGCATAGAGCGTGGGAGG
VDAG_08591-up-R	GGACTTAAUCGATGTGGAGGTGAGGAGA<br>C
VDAG_08591-down-F	GGCATTAAUTTACACGCCAAAGCACAAG

VDAG_08591-down-R	GGTCTTAAUCAGGGAGAGCTCCGACAG
VDAG_10179-up-F	GGGTTTAAUGTTGGAGCTGAGATGCCTG
VDAG_10179-up-R	GGACTTAAUCGTGGGACGAGATGGTTTG
VDAG_10179-down-F	GGCATTAAUCGCTTCCTGCTATGGTCTG
VDAG_10179-down-R	GGTCTTAAUAGCTCGCTGGTGGTTGTCG
VDAG_05405-up-F	GGGTTTAAUCGACGATTCCACTTCACG
VDAG_05405-up-R	GGACTTAAUGCCATGATCCTTTGCTTG
VDAG_05405-down-F	GGCATTAAUGCTCGTGAAGCTATAATGG
VDAG_05405-down-R	GGTCTTAAUTCACCGTAATAGAGGGTCG
VDAG_00376-up-F	GGGTTTAAUCTTGCTATGTCGGTGGAG
VDAG_00376-up-R	GGACTTAAUTTGATGACGCTCTCACAG
VDAG_00376-down-F	GGCATTAAUGCAGCGGACGGTATGATG
VDAG_00376-down-R	GGTCTTAAUGTTGGAGTGGCCGATTAG
VDAG_00376-UUP	TGTGACCTTGTTGTGATGGTCTGC
VDAG_00376-DDN	TGCGTATGTGAAGTGGATGGAAGG
VDAG_05405-UUP	CGGTAAGGCAGAAGTAGTATGATC
VDAG_05405-DDN	GGCTTCGTCTTCAGTCAGGTTGG
VDAG_10179-UUP	CTCGTCTGCATCAGCCACGTGC
VDAG_10179-DDN	TGCTACTACGCAGCCTACAATGC
VDAG_00419-UUP	TGATGATCTGCAGTGAGAACGAGG
VDAG_00419-DDN	CAGTCACAGCAACGCTGGATGG
VDAG_00420-UUP	CCTGGTCCACACGTTGCGATCC

VDAG_00420-DDN	GCCGACGAGTGGACCTACGAC
VDAG_02580-UUP	GGCTGCAGATTAGTGCATGTCC
VDAG_02580-DDN	ACGAACCTCACCACCATCAACAAC
VDAG_03141-UUP	GGATACTTAGCAGGTACCTGAGC
VDAG_03141-DDN	CTCTTGCACAGGCCTGACTAGC
VDAG_08591-UUP	TCAGGTAGGAGGCAGGTATTCG
VDAG_08591-DDN	AGTTCAACACAGGCTTCCAGTCC
VdKO_5'check_R	AAATTTTGTGCTCACCGCCTGGAC
VdKO_3'check_F	TCTCCTTGCATGCACCATTCCTTG
VDAG_00376-FS-F	CAGTGCATCATGTCTGACG
VDAG_00376-FS-R	CCTGTCGCCAAACTACGC
VDAG_05405- FS-F	CATACCAGAAGTTCATCTTC
VDAG_05405- FS-R	TGCTAGAACCCATATCGAG
VDAG_10179- FS-F	CTCGTCCCACGATCCTAC
VDAG_10179- FS-R	CAGACCATAGCAGGAAGC
VDAG_00419- FS-F	ACACCACCTGATCTTATCC
VDAG_00419- FS-R	TGGAGTGCAGAGGTTACG
VDAG_00420- FS-F	CTCGTCTGCTTCAAGTCC
VDAG_00420- FS-R	GAGTCGTTGCAATGCTGC
VDAG_02580- FS-F	ACGATTTCTACAACGACCG
VDAG_02580- FS-R	AATCGAAAAGGAGGAGACC
VDAG_03141- FS-F	AGCTGACTCTGTTACTTCC

VDAG_03141- FS-R	TCGCTGTCAACACCAAGC
VDAG_08591- FS-F	CAATAAACCTCCTCGAACC
VDAG_08591- FS-R	GCTTTGGCGTGTAAGACC
