# Supplementary figures and images for: Chitin Synthase Genes Are Differentially Required for Growth, Stress Response, and Virulence in Verticillium dahliae
Source: J Fungi (Basel). 2022 Jun 28;8(7):681. doi: 10.3390/jof8070681 (PMC9320267; doi:10.3390/jof8070681)

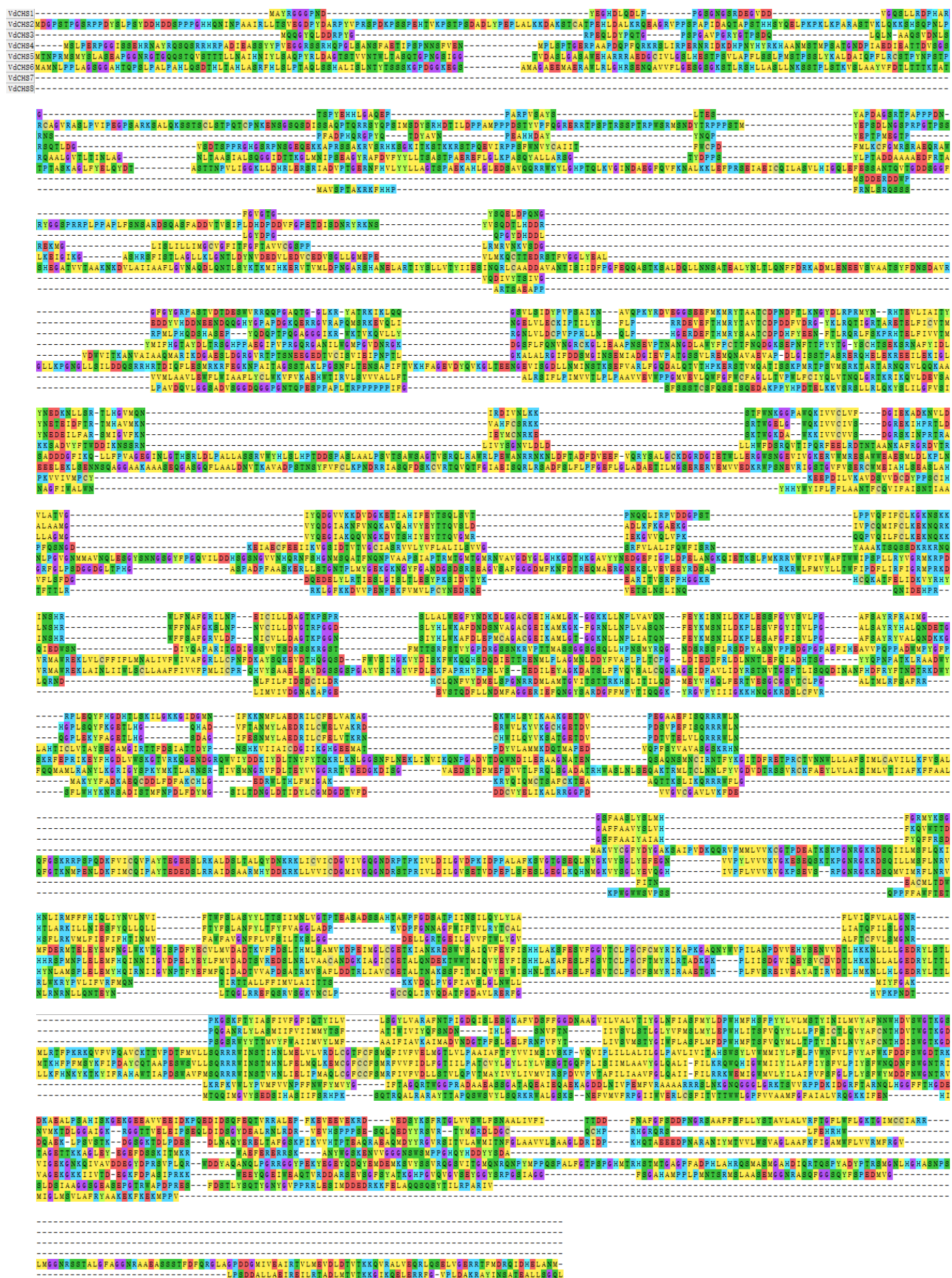

Figure S1. The amino-acid alignment of eight chitin synthases in *Verticillium dahliae*

Supplement: Supplementary file 1 [file jof-08-00681-s001.zip › Supplementary Figures andTable/Fig S1 final.pdf]
